# Real-world outcomes in hereditary angioedema: first experience from the Icatibant Outcome Survey in the United Kingdom

**DOI:** 10.1186/s13223-018-0253-x

**Published:** 2018-08-06

**Authors:** Hilary J. Longhurst, John Dempster, Lorena Lorenzo, Matthew Buckland, Sofia Grigoriadou, Christine Symons, Claire Bethune, Vincent Fabien, Catherine Bangs, Tomaz Garcez

**Affiliations:** 1 Department of Clinical Biochemistry and Immunology, Addenbrooke’s Hospital, Cambridge University Hospitals NHS Foundation Trust, Cambridge, UK; 2 Department of Immunology, Barts Health NHS Trust, London, UK; 3 Department of Immunology, Plymouth Hospitals NHS Trust, Plymouth, UK; 4 Shire, Zug, Switzerland; 5 Department of Immunology, Central Manchester University Hospital NHS Foundation Trust, Manchester, UK

**Keywords:** C1-inhibitor deficiency, Acquired angioedema, Hereditary angioedema, Icatibant, Icatibant Outcome Survey

## Abstract

**Background:**

Hereditary angioedema (HAE) is a potentially life-threatening, bradykinin-mediated disease, often misdiagnosed and under-treated, with long diagnostic delays. There are limited real-world data on best-practice management of HAE in the UK.

**Objectives:**

To characterize the clinical profile, management and outcomes of patients with HAE type I and II from three specialist centres in the UK using data from the Icatibant Outcome Survey (IOS; Shire, Zug, Switzerland), an international observational study monitoring safety and effectiveness of icatibant, a selective bradykinin B2 receptor antagonist.

**Methods:**

We performed retrospective analyses of IOS data for patients with HAE type I and II from three centres in the UK and compared UK data with pooled IOS data from 10 countries (48 centres).

**Results:**

Analyses included 73 UK and 579 non-UK patients with HAE type I or II. Median diagnostic delay was 6.2 and 5.9 years, respectively. Analysis of data collected from February 2008 to July 2016 included 286 icatibant-treated attacks in 58 UK patients and 2553 icatibant-treated attacks in 436 non-UK patients (median of 3.0 attacks per patient in both groups). More attacks were treated by icatibant self-administration in UK patients (95.8%) than in non-UK patients (86.8%, p < 0.001). Time to icatibant treatment, time to resolution and attack duration were not significantly different in the UK versus non-UK patients.

**Conclusion:**

UK patients from the specialist centres studied report similar diagnostic delay and similar icatibant treatment outcomes to their non-UK counterparts. However, improvements in the timely diagnosis of HAE are still required.

*Trial registration* ClinicalTrials.gov NCT01034969

## Background

Hereditary angioedema (HAE) due to C1 inhibitor (C1-INH) deficiency is a rare autosomal dominant disease caused by mutations in the *SERPING1* gene resulting in reduced levels (type I) or dysfunction (type II) of C1-INH. HAE type I and II attacks are characterized by recurrent swelling, commonly occurring in the skin, abdomen and larynx, which can be severe and debilitating [1, 2]. In the UK, there are an estimated 1500 people with C1-INH deficiency, many of whom are undiagnosed [3]. A number of consensus international and UK guidelines regarding the diagnosis and management of HAE have been published [4–9]. However, the rarity of HAE means that non-specialist physicians are often unfamiliar with these conditions and patient management is usually conducted in coordination with tertiary centres, with particular emphasis on patient self-care [3, 9].

In the UK, intravenously administered plasma-derived C1-INH (Berinert^®^, Cinryze^®^), recombinant C1-INH (Ruconest^®^) and the subcutaneously administered bradykinin receptor antagonist icatibant (Firazyr^®^) are licensed and effective for the treatment of acute attacks of HAE and are now commissioned nationally for home-based self-administration [10–15]. Following the initial approval of icatibant within Europe, the Icatibant Outcome Survey (IOS), a real-world patient registry documenting the clinical outcomes of patients treated with icatibant, was established to fulfil a European medicines agency post-marketing surveillance safety requirement. A number of clinical registries and surveys for HAE have been conducted in several countries [16–19], which may be used to support quality improvement activities. Although a recent UK national audit has helped to characterize the experience of patients with HAE [20], there is a relative paucity of UK data to help improve clinical practice and better understand the burden of disease.

This report summarizes observational UK data collected within the first 6 years of IOS (March 2010–July 2016) to characterize the clinical profile, management and outcomes of patients with HAE. These data could be used as an unofficial ‘benchmark’ of standards from three major centres within the UK that offer specialist management and up-to-date treatment protocols. In addition, data from the UK are compared with other IOS countries, with the aim of identifying potential areas for quality improvements.

## Methods

### Study design and patients

IOS (NCT01034969) is a prospective, international, observational study; study methodology has been published elsewhere [21]. The registry enrols patients with C1-INH deficiency (HAE types I and II), consistent with the European approval conditions for icatibant, but any patient prescribed subcutaneous icatibant treatment is eligible for inclusion. IOS is conducted in accordance with the Declaration of Helsinki and the international conference on harmonisation good clinical practice. Approval was obtained from ethics committees and/or local health authorities at all centres. Written, informed consent was obtained from all enrolled patients aged ≥ 18 years (≥ 16 years in the UK); consent was provided by parents/legal guardians for patients aged < 18 years (< 16 years in the UK).

The analyses described herein are based on IOS data collected between July 2009 and July 2016 for patients with HAE type I or II; patients with other forms of angioedema were excluded from this analysis. Retrospective data for attacks recorded prior to IOS enrolment were also collected, dating back to February 2008. During this period, a total of 51 centres in 11 countries contributed data for this analysis: Austria (*n* = 1), Brazil (n = 1), Germany (*n* = 7), Denmark (*n* = 1), Spain (*n* = 9), France (*n* = 17), Greece (*n* = 2), Israel (*n* = 4), Italy (*n* = 5), Sweden (*n* = 1) and the UK (*n* = 3). We also include a separate description of seven patients with acquired angioedema (AAE) due to C1-INH deficiency for this period from the UK sample, including one patient who was treated with icatibant for acute attacks (unlicensed indication).

**Fig. 1:**
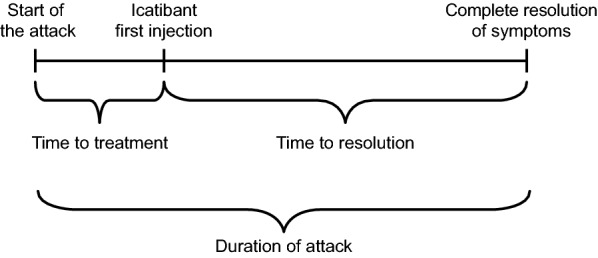
Schematic of effectiveness measures used in the Icatibant Outcome Survey

Patient demographics and characteristics were recorded at enrolment (IOS entry [baseline]), including information on HAE attacks (including icatibant-treated) before IOS entry. Information relating to physical examination, icatibant treatment, concomitant medications and adverse events was collected at enrolment and at routine visits, recommended every 6 months thereafter. Data for attacks treated with icatibant or untreated attacks were recorded, including rescue medication and long-term prophylaxis usage; data for attacks treated with other products are not collected in IOS. For each icatibant-treated HAE attack, data were collected for time to treatment (time between the start of the attack and the first icatibant injection), duration of attack (time between onset of attack and complete resolution of symptoms) and time to resolution (time between first icatibant injection and complete resolution of symptoms) (Fig. 1). Data recorded for untreated attacks included frequency and duration of untreated attacks in the prior 12 months and potential reasons for non-treatment. The IOS protocol does not specify how patients should record attacks. Patients used several methods to record attacks, including diaries, evaluation forms and, in some cases, reporting attacks contemporaneously to specialist nurses. Self-administration of treatment included administration by family members or the patient, according to national prescribing information, after training in subcutaneous injection technique by a healthcare professional. Attack severity was described by patients and was verified by a healthcare professional at routine visits. Severity was classified as: none (absence of symptoms); mild (mild interference with daily activities); moderate (moderate interference with daily activities and no other treatment required; severe (severe interference with daily activities and with or without other treatment); and very severe (very severe interference with daily activities and other treatment required) [22].

### Statistical analyses

Results of UK versus non-UK populations were compared. A mixed-model analysis of repeated measures (Proc Mixed; SAS Institute, Cary, NC, USA) was used to compare time to treatment, time to resolution and duration of attack. The Chi squared test was used for the comparison of dichotomous data, with a statistical significance level of alpha = 0.05. Data are presented as median (interquartile range [IQR]) or mean (standard deviation [SD]), unless otherwise specified.

## Results

### Patient characteristics

Between July 2009 and July 2016, 652 patients diagnosed with HAE type I or II were entered into the IOS database. Of these, 73 patients were from three centres in the UK (enrolled from March 2010 onwards) and 579 patients were from 48 centres outside the UK. Approximately 60% of patients in both UK and non-UK groups were female. Median patient age at extract was 42.2 and 43.3 years for UK and non-UK patients, respectively. Patient demographics and baseline characteristics are presented in Table 1. In the UK, one patient received icatibant under the age of 18 years (two icatibant injections, one each for two separate attacks at 17.6 and 17.8 years). Outside the UK, five patients received icatibant injections under the age of 18 years (range 16.2–17.9 years). Of these five non-UK patients, two patients each received a single icatibant injection, one patient received three icatibant injections for three separate attacks, one patient received four icatibant injections for four separate attacks and one patient received ten icatibant injections for ten separate attacks.

**Table 1:** Patient demographics and baseline characteristics

Characteristic	Patients with HAE type I or II	
	UK (*N* = 73)	Non-UK (*N* = 579)
Age at extract, years		
Mean (SD)	42.9 (14.7)^a^	45.1 (15.2)^b^
Median (range)	42.2 (18–82)^a^	43.3 (6–86)^b^
Gender, *n* (%)		
Male	29 (39.7)	235 (40.6)
Female	44 (60.3)	344 (59.4)
Age at first symptoms, years		
Mean (SD)	11.3 (9.5)^c^	14.1 (11.2)^d^
Median (IQR)	10.0 (5.0, 16.0)^c^	13.0 (6.0, 19.0)^d^
Age at diagnosis, years		
Mean (SD)	21.5 (12.7)^e^	24.6 (15.9)^f^
Median (IQR)	18.9 (12.6, 30.0)^e^	21.0 (13.2, 34.2)^f^
Delay between first symptoms and diagnosis, years		
Mean (SD)	9.5 (13.9)^c^	10.5 (13.6)^g^
Median (IQR)	6.2 (0.0, 17.5)^c^	5.9 (0.4, 17.6)^g^
Employment status, *n* (%)^h^		
Employee	46 (63.0)	218 (52.0)
Self-employed	7 (9.6)	15 (3.6)
Homemaker	3 (4.1)	20 (4.8)
Leave of absence/sabbatical	1 (1.4)	2 (0.5)
Pre-school	0	4 (1.0)
Retired	3 (4.1)	42 (10.0)
Student	8 (11.0)	53 (12.6)
Unemployed	4 (5.5)	34 (8.1)
Other/unknown	4 (5.5)	43 (10.3)

*HAE* hereditary angioedema, *IQR* interquartile range, *SD* standard deviation

^a^*n* = 73

^b^*n* = 579

^c^*n* = 58

^d^*n* = 497

^e^*n* = 70

^f^*n* = 535

^g^*n* = 486

^h^Patients could have more than one employment status (UK sample *n* = 73, non-UK patients *n* = 419)

### Socioeconomic data

**Fig. 2:**
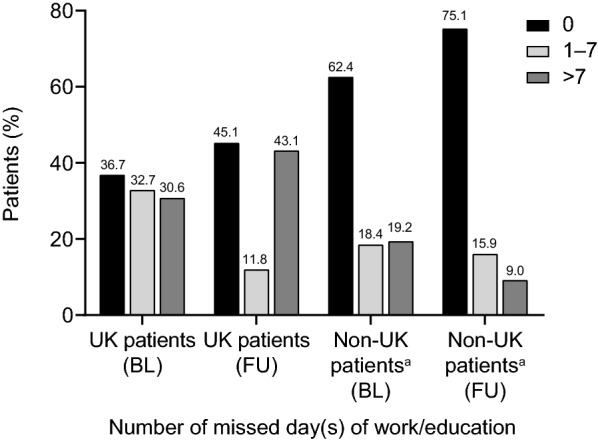
Proportion of the UK and non-UK IOS patients with days of missed work or education prior to IOS entry and during the IOS observation period. For the UK and non-UK patients, respectively, *n* = 49 and *n* = 125 for the period before IOS entry and *n* = 51 and *n* = 201 for the IOS follow-up period. *BL* baseline (12 months prior to IOS entry), *FU* follow-up, *IOS* Icatibant Outcome Survey. ^a^Non-UK countries are Austria, Brazil, Denmark, France, Germany, Greece, Israel, Italy, Spain and Sweden

**Fig. 3:**
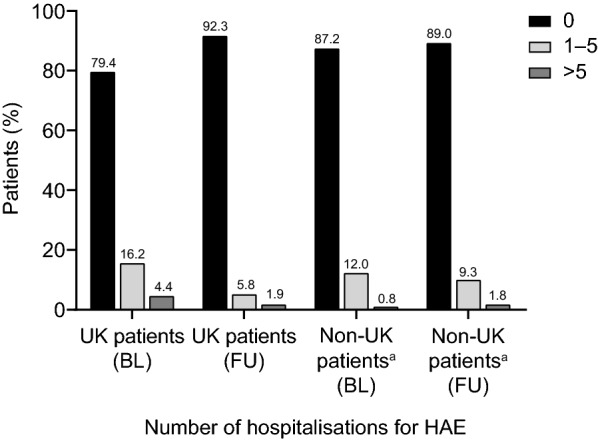
Proportion of the UK and non-UK IOS patients hospitalized at baseline (prior to IOS entry) and during IOS observation. For the UK and non-UK patients, respectively, *n* = 68 and *n* = 367 for the period before IOS entry and *n* = 52 and *n* = 399 for the IOS follow-up period. *BL* baseline (12 months prior to IOS entry), *FU* follow-up, *IOS* Icatibant Outcome Survey. ^a^Non-UK countries are Austria, Brazil, Denmark, France, Germany, Greece, Israel, Italy, Spain and Sweden

Employment status for UK and non-UK patients at IOS entry is shown in Table 1. In the UK, a significantly higher proportion of patients were employed or self-employed compared with non-UK patients; 53/73 (72.6%) versus 233/419 (55.6%); p = 0.0111. The proportion of students among the UK and non-UK patients was similar: 8/73 (11.0%) and 53/419 (12.6%), respectively. More patients in the UK missed work or education prior to IOS compared with non-UK patients (63.3% versus 37.6%, respectively; p = 0.009) and during the IOS observation period (54.9% versus 24.9% respectively, p < 0.001) (Fig. 2). The proportion of patients missing days of work or education improved in the non-UK patients during the IOS observation period (decreasing from 37.6 to 24.9%) The proportion of patients missing days of work or education showed a non-significant trend to improvement in the UK during the IOS observation period (decreasing from 63.3 to 54.9%, p = 0.060); however the reduction was seen only in patients reporting absences of ≤ 7 days. More patients in the UK required hospitalisation prior to IOS compared with non-UK patients (20.6% versus 12.8%, respectively; p = 0.037), however no difference in rates of hospitalisation during the IOS observation period between UK and non-UK patients was observed (p = 0.696) (Fig. 3). However, within the UK the rate of hospitalisations prior to IOS compared to the IOS observation period showed a trend to improvement but this did not reach statistical significance (p = 0.098).

### Use of long-term prophylaxis

In UK and non-UK patients, respectively, ongoing long-term prophylaxis use at IOS entry and during the follow-up period was 75.3% (55/73) and 44.4% (257/579). Attenuated androgens (danazol, stanozolol and oxandrolone) were the most commonly used ongoing long-term prophylaxis medication (at IOS entry and during the follow-up period) by both UK and non-UK patients (accounting for 59.9 and 66.9% of usage, respectively). In the UK, attenuated androgens were used as ongoing long-term prophylaxis in 54.5% (18/33) of patients from the Barts Health NHS Trust, London; 71.4% (10/14) of patients from the Central Manchester University Hospital NHS Foundation Trust, Manchester and 62.5% (5/8) of patients from Plymouth Hospital NHS Trust. Other ongoing long-term prophylactic agents used in the UK overall were C1-INH (7.3%; 4/55) and tranexamic acid (23.6%, 13/55).

### Delay in diagnosis

**Fig. 4:**
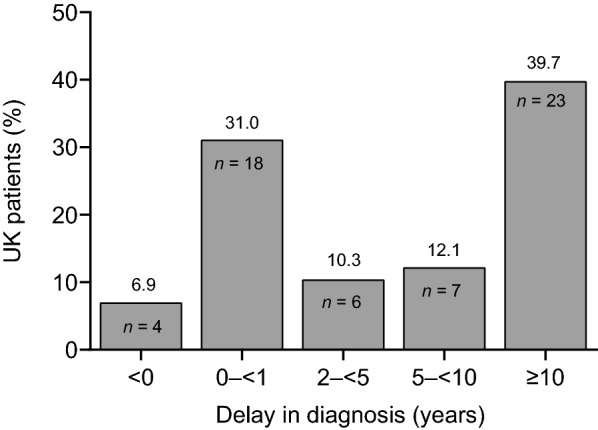
Proportion of HAE patients in the UK sample with a delay in diagnosis. *HAE* hereditary angioedema. *n* = 58

Median age at diagnosis of HAE type I or II was 18.9 years for the UK patients and 21.0 years for non-UK patients, with a median delay between first symptoms and diagnosis of 6.2  and 5.9 years, respectively (Table 1). Of 58 patients in the UK with available data, 36 (62.1%) patients experienced a delay in diagnosis of ≥ 2 years, with 23 (39.7%) patients having a delay of ≥ 10 years (Fig. 4). There was a clear biphasic distribution in time from symptoms to diagnosis.

### Treatment of HAE attacks with icatibant

#### Attack rate

Among 73 patients in the UK, 58 (79.5%) had reported at least one attack treated with icatibant, with a total of 286 icatibant-treated attacks. These attacks were derived from 31 patients from the Barts Health NHS Trust, London, 16 patients from the Central Manchester University Hospital NHS Foundation Trust, Manchester and 11 patients from the Plymouth Hospitals NHS Trust, Plymouth (Table 2). A total of 2553 icatibant-treated attacks were reported for 436/579 (75.3%) patients in countries outside the UK. A median of 3.0 attacks per patient were treated with icatibant in both the UK and outside the UK (Table 3).

**Table 2:** HAE type I or II attacks treated with icatibant in the UK sample

IOS period	Barts health NHS trust, London *N* = 31		Central Manchester university hospital NHS foundation trust, Manchester *N* = 16		Plymouth hospitals NHS trust *N* = 11		All UK sample *N* = 58	
	Patients, *n*	Attacks, *n*	Patients, *n*	Attacks, *n*	Patients, *n*	Attacks, *n*	Patients, *n*	Attacks, *n*
Before IOS entry	23	40	11	84	11	21	45	145
After IOS entry	24	146	12	124	5	20	41	290
Total	31	186	16	208	11	41	58	435

*HAE* hereditary angioedema, *IOS* Icatibant Outcome Survey

**Table 3:** Comparison of HAE attacks treated with icatibant and untreated attacks

Parameter	Patients with HAE type I or II	
	UK *N* = 73	Non-UK *N* = 579
Icatibant-treated attacks^a^		
Patients treated with icatibant, *n*	58	436
Attacks treated with icatibant per patient		
Mean (SD)	7.5 (12.8)	7.6 (12.5)
Median (IQR)	3.0 (1.0, 6.0)	3.0 (1.0, 8.0)
Untreated attacks in year prior to IOS entry^b^		
Patients with at least one untreated attack, *n*	46^c^	219^d^
Untreated attacks per patient		
Mean (SD)	8.5 (16.8)	7.4 (14.0)
Median (IQR)	2.0 (0, 5.0)	2.0 (0, 8.0)
Untreated attacks in the IOS observation period^a^		
Patients with at least one untreated attack, *n*	30^e^	198^f^
Untreated attacks per patient		
Mean (SD)	10.9 (23.2)	8.2 (17.3)
Median (IQR)	2.0 (0.0, 8.0)	1.0 (0, 8.0)

*HAE* hereditary angioedema, *IOS* Icatibant Outcome Survey, *IQR* interquartile range, *SD* standard deviation

^a^Attacks in year prior to IOS entry and through the IOS observation period

^b^Untreated attacks were defined as attacks not treated with icatibant or any other treatment

^c^19 patients had no untreated attacks

^d^114 patients had no untreated attacks

^e^22 patients had no untreated attacks

^f^150 patients had no untreated attacks

#### Icatibant-treated versus untreated attacks

In total, 854 attacks were recorded after IOS entry for the 73 UK patients. Of these, 286 (33.5%) attacks were treated with icatibant and 568 (66.5%) remained untreated. As IOS is an icatibant registry, data for attacks treated with C1-INH were not recorded. For non-UK patients, 5423 attacks were recorded after IOS entry, with 2553 (47.1%) attacks treated with icatibant and 2870 (52.9%) attacks not treated with any treatment. In UK patients, the median number of untreated attacks per patient was the same (2.0) in the IOS observation period (data collected at routine visits) as in the period prior to IOS entry (data collected at baseline) (Table 3). For non-UK patients, the median number of untreated attacks per patient was lower (1.0 versus 2.0, respectively) in the IOS observation period than in the period prior to IOS entry. Since C1-INH-treated attacks were not recorded, no conclusion can be drawn as to overall percentages. However, it remains clear that a substantial numbers of attacks are untreated and that in the UK, these outnumber those treated with icatibant by almost 2–1. Although we do not have data on severity or complete data on possible reasons for non-treatment, the duration of untreated attacks, both prior to IOS enrolment (median 72 h for both UK and non-UK patients) and IOS follow-up period (72 versus 79 h for the UK and non-UK patients, respectively) is substantially longer than for treated attacks (9.0 versus 8.6 h; Table 4).

**Table 4:** Time to treatment, time to resolution and duration of icatibant-treated HAE attacks

Endpoint	Patients with HAE type I or II						
	UK (*N* = 73)			Non-UK (*N* = 579)			p value^b^
	*n* ^a^	Mean (SD)	Median (IQR)	*n* ^a^	Mean (SD)	Median (IQR)	
Time from attack onset to treatment, h^c^	222	2.9 (4.8)	0.8 (0.4, 3.0)	1120	3.9 (7.3)	1.3 (0.5, 4.0)	0.0632
Time to complete symptom resolution, h^d^	222	10.5 (14.1)	6.0 (1.3, 14.0)	1120	11.8 (16.1)	5.8 (2.0, 14.1)	0.2774
Duration of attack, h^e^	222	13.4 (15.4)	9.0 (2.5, 18.5)	1120	15.6 (18.9)	8.6 (4.0, 20.0)	0.1022

*HAE* hereditary angioedema, *IQR* interquartile range, *n* number of evaluable attacks, *SD* standard deviation

^a^Attacks with complete data for time to treatment, time to complete resolution and attack duration, excluding attacks treated > 100 h after attack onset

^b^Mixed-model analysis of repeated measures comparing the UK versus non-UK IOS datasets

^c^Time between the start of the attack and the first icatibant injection

^d^Time between first injection of icatibant and complete resolution of symptoms

^e^Time between start of attack and complete resolution of symptoms

#### Severity of attacks

In the UK, 65.5% (279/426) of attacks before treatment were classified (where data were available) as severe/very severe; 26.1% (111/426) as moderate; and 8.5% (36/426) as mild or very mild. For non-UK patients, attack severity before treatment was classified as severe/very severe, 53.3% (1441/2704 attacks); moderate, 37.2% (1005/2704 attacks); and mild or very mild, 9.5% (258/2704 attacks). No difference in attack severity was observed between UK and non-UK patients (p = 0.1724).

#### Use of icatibant self-administration

**Fig. 5:**
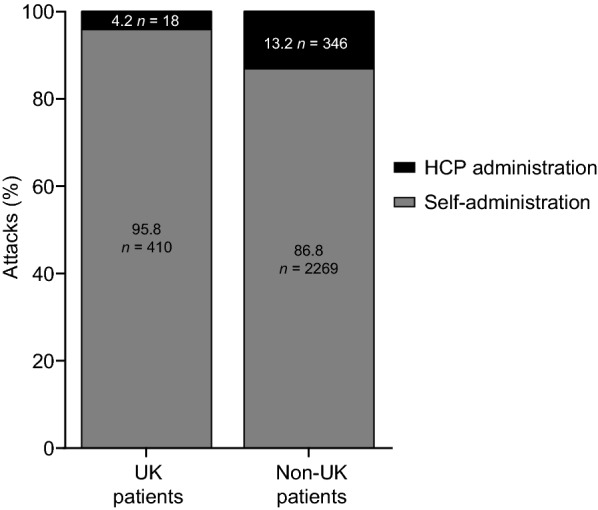
Proportion of HAE attacks treated with icatibant by self-administration or administration by HCPs per attack in the UK and non-UK patients. *HAE* hereditary angioedema, *HCP* healthcare professional

**Fig. 6:**
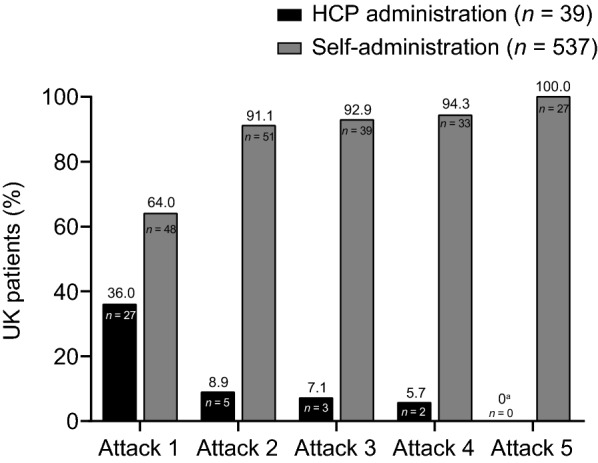
Administration of icatibant for first five HAE attacks in UK patients. *HCP* health care professional. ^a^The remaining two HCP-administered instances of icatibant occurred during attack 14 (9.1%) and attack 19 (11.1%). Family member administrations are included in the self-administration category. All non–self-administrations are included in the HCP category. Missing attack dates are not taken into account

Of the attacks treated with icatibant, the majority were treated by self-administration both inside and outside the UK (95.8% versus 86.8%, respectively) (Fig. 5) with the rate of self-administration in the UK significantly higher (p < 0.001) compared to non-UK patients. Healthcare professionals administered icatibant for first attacks in around one-third of UK and non-UK patients in the IOS observation period (16/57 [28.1%] and 133/366 [36.3%], respectively). The mode of icatibant administration for the first five attacks for UK patients is shown in Fig. 6.

### Time to icatibant treatment, time to resolution and attack duration

**Fig. 7:**
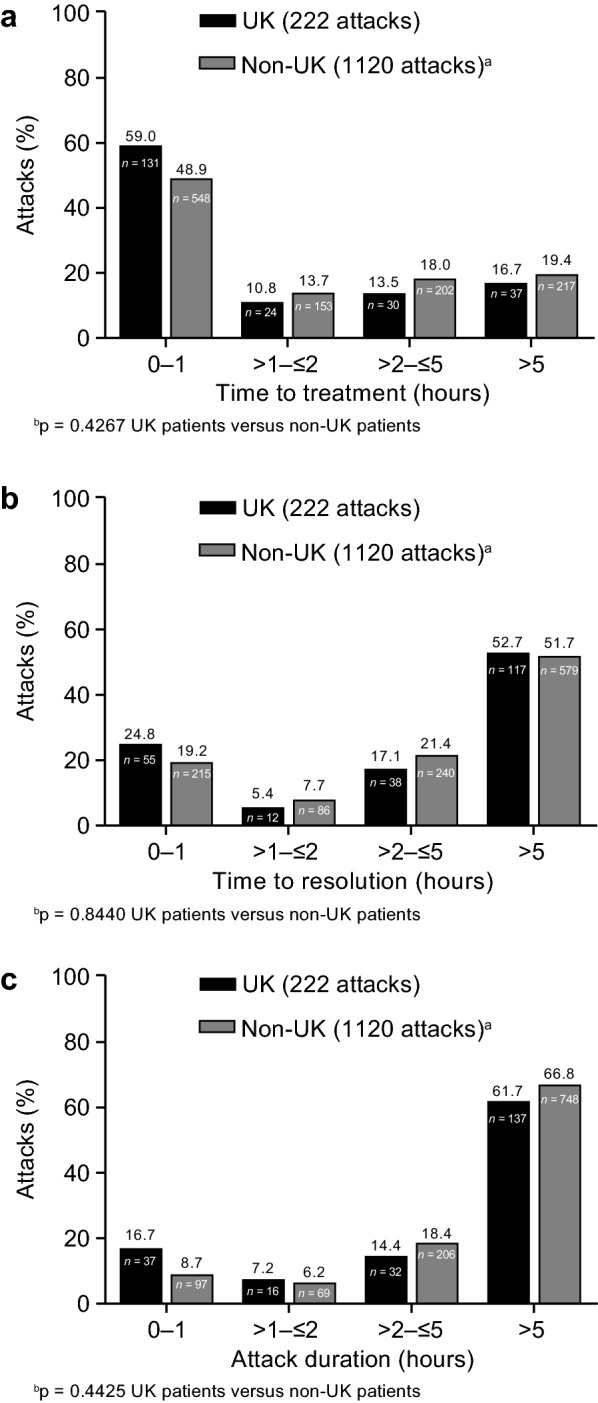
Proportion of icatibant-treated HAE attacks (with complete data for time to treatment, time to resolution and attack duration) in UK patients and non-UK patients according to **a** time to treatment, **b** time to symptom resolution and **c** duration of attack. *HAE* hereditary angioedema, *Time to treatment* time between the start of the attack and the first icatibant injection, *Time to resolution* time between first injection of icatibant and complete resolution of symptoms, *Attack duration* time between start of attack and complete resolution of symptoms. ^a^Non-UK countries are Austria, Brazil, Denmark, France, Germany, Greece, Israel, Italy, Spain and Sweden. ^b^Generalized linear model for repeated measures

Time to treatment with icatibant, time to resolution and duration of attack were not significantly different in the UK compared with non-UK patients, although there was a trend to earlier treatment in UK patients, p = 0.0632, p = 0.2774 and p = 0.1022, respectively (Table 4, Fig. 7).

#### Use of C1-INH rescue

In the UK, excluding one outlier with a very atypical pattern of use (described below), C1-INH was used as rescue medication in 12.7% of icatibant-treated attacks (48/378 attacks in 15/57 patients) compared with 9.2% of icatibant-treated attacks (304/3306 attacks in 86/436 patients) in non-UK patients.

#### Clinical experience: patient with high icatibant reinjection and C1-INH rescue medication usage

**Fig. 8:**
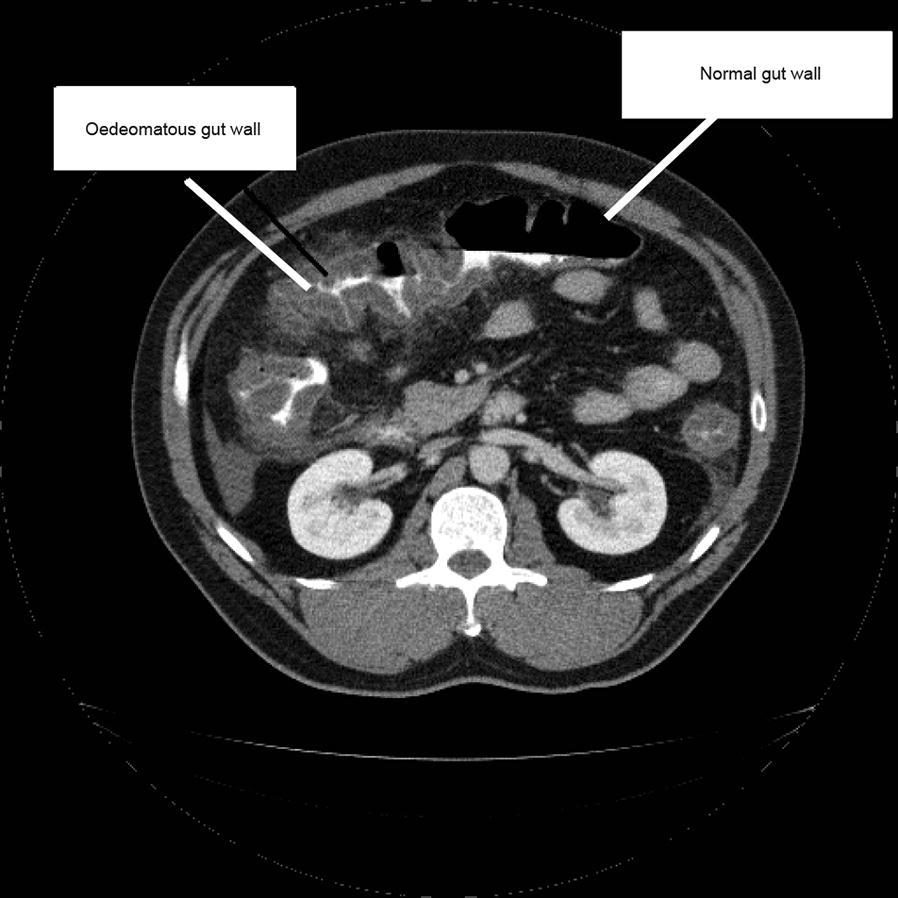
Abdominal computed tomography image obtained at admission of a 47-year-old male patient with HAE type I with abdominal angioedema

Further analysis of the pattern of icatibant usage revealed that one patient in the UK had a high rate of icatibant reinjection combined with high usage of C1-INH rescue medication. This 50-year-old man with HAE type I self-administered icatibant for 57 HAE attacks (very severe, *n* = 12; severe, *n* = 39; and moderate, *n* = 6) between 22 October 2009 and 25 October 2014. Approximately half (27/57) of these attacks were treated with a second injection of icatibant and 86.0% (49/57) with C1-INH rescue medication. The median (IQR) time to first icatibant injection was 0.5 (0.5–1.0) h, median (IQR) time to complete resolution was 1.0 (0.5–9.5) h and median (IQR) duration of attack was 3.5 (1.0–11.0) h. Median (IQR) time between first and second icatibant injection was 9.0 (6.0–12.0) h and the median (IQR) time between second injection and complete resolution was 0.5 (0.5–0.5) h. During the treatment period the patient was under intense psychological stress and had frequent bouts of confirmed abdominal angioedema (Fig. 8). The majority of icatibant-treated attacks (68.4%; 39/57) were abdominal. The patient was receiving long-term danazol prophylaxis at attack onset for 49.1% (28/57) of icatibant-treated attacks. However, in spring 2011 prophylaxis with danazol became contraindicated owing to the development of abnormal liver function tests, hyperlipidaemia and weight gain. The patient initiated long-term prophylaxis with C1-INH in April 2011 and has reported only three icatibant-treated HAE attacks since that date.

#### Acquired angioedema

Seven patients from the UK and 34 non-UK patients had a diagnosis of AAE due to C1-INH deficiency. Icatibant is not currently licensed for use in patients with AAE due to C1-INH deficiency and these patients were not included in the analysis described above. However, for completeness of the UK dataset, we include a brief description of the seven UK patients in Table 5 [23].

**Table 5:** UK patients with acquired angioedema

Patient no.	Gender	Age at IOS enrolment, years	Icatibant-treated angioedema attacks^a^, *n*	HCP-administered icatibant injections, *n*	Self-administered icatibant injections, *n*
1	Male	50.5	53	3	51
2	Male	63.1	27	6	22
3	Female	49.7	2	1	1
4	Male	70.9	1	–	1
5	Female	28.6	0	–	–
6	Female	59.8	0	–	–
7	Female	38.4	0	–	–

*HCP* healthcare professional, *IOS* Icatibant Outcome Survey

^a^July 2009 to July 2016

## Discussion

IOS has allowed the follow-up of patients with HAE type I/II in the UK and centres across Europe, the Middle East and South America. These data from three UK centres suggest that HAE remains under-recognized in the UK, as shown by long diagnostic delays. Whilst some patients were diagnosed prior to first symptoms (presumably based on family history) or at the time of or soon after first symptoms, others experienced very long delays, with consequent increased risk of avoidable mortality and morbidity. The delay in diagnosis in the UK (*n* = 73) was comparable to that in non-UK countries, which supersedes the previously reported trend from a smaller IOS UK patient population (*n* = 12) [24]. The delay in diagnosis in the UK was shorter than the delay in diagnosis recently reported in a UK National Audit of HAE; however this may be explained by differences in methodology, as the authors of the UK National Audit excluded all patients who received diagnoses before experiencing symptoms [20]. The overarching message from all these studies is that delay in diagnosis is highly variable; a recent IOS analysis found approximately 50% of patients with HAE type I/II had received a prior misdiagnosis, most commonly allergic angioedema or appendicitis [25]. Increased awareness of HAE could reduce time to diagnosis and permit appropriate management of the condition, thus reducing the morbidity and mortality associated with undiagnosed HAE [26].

Self-administration of HAE treatment at home offers the potential for earlier treatment and symptom control [21], reducing the impact of HAE on physical, social and economic well-being and reducing healthcare resource use [8, 27, 28]. The UK patients included a higher proportion of attacks treated by self-administration compared with non-UK patients (95.8% versus 86.8%, respectively). This may partially be due to the later enrolment of patients in the UK, with 66/73 (90.4%) patients joining the study in 2011 or later, compared with a larger number of patients in Europe (177/579 [30.6%]) who enrolled up to 2 years prior to the 2011 change in indication to include self-administration. Importantly, each of our UK centres in IOS is a strong advocate of home therapy and patient empowerment, in accordance with UK government policy and home therapy guidance [3, 7, 10]. There is long-standing experience of treating and training patients for self-administration in the UK, compared with more recent experience in some other European countries [15, 29]. This is greatly facilitated by the availability of immunology specialist nurses who develop considerable expertise in self-administration training and patient support [14, 29, 30]. Although a higher proportion of UK patients self-administered icatibant compared with non-UK patients, the proportion of first attacks treated by self-administration was similar for the UK sample and non-UK patients (71.9% versus 63.7%, respectively). It is recommended that the first dose of icatibant is given under the supervision of a healthcare professional to ensure adequate training [12] and allow monitoring of tolerability to the drug immediately after administration. However, the need for professional supervision can be a barrier to accessing treatment and therefore remains a recommendation rather than a requirement. These IOS data support our clinical experience that patients tolerate icatibant well and, with encouragement and support, rarely require more than one training session. Moreover, the ease of use of icatibant has enabled a greater proportion of patients to self-administer treatment for HAE attacks than is possible with C1-INH treatment, which requires intravenous access.

Time to treatment, time to resolution and duration of attack were not significantly different in the UK compared with non-UK patients. Previously, using a data extract of April 2015 (presented at UK PIN meeting 2015), we had reported that UK IOS patients experience a significantly shorter time to treatment, time to resolution and attack duration than patients from other IOS countries. However, the July 2016 data presented herein clearly show this gap has closed, and a recent country comparison of IOS data provides additional information regarding regional variations in both delay in diagnosis and icatibant use across six EU member states [31]. Previous IOS analyses have demonstrated that earlier treatment can reduce both overall attack duration and time to resolution [21].

Patients with HAE suffer impaired quality of life and incapacity, with time away from school, work, social events and family life, and reduced productivity [2, 32–39]. A higher proportion of the UK patients were employed or in education compared with non-UK patients. In the UK, a higher percentage (72.6%) were employed or self-employed compared with the UK national audit of HAE (48% in paid employment) [20]. As IOS is an icatibant registry, it may be that factors that lead to icatibant prescription result in a degree of selection bias, thereby giving rise to the differences between this population and the wider group of UK patients with HAE. However, the data could also be consistent with the early adoption of icatibant by the specialist centres studied and the inclusion of data on patients after 2013, when barriers to icatibant prescription were relaxed in the UK following adoption of a policy of central funding [8]. Patients in the UK missed more time from work owing to HAE than non-UK patients. Despite needing to miss more work for HAE attacks, a higher percentage of UK patients were able to maintain employment, perhaps because more patients self-treated with icatibant. This suggests that an effective self-administered treatment for angioedema attacks may be an important element in enabling patients to stay in work [8]. For example, one of the patients with AAE due to C1-INH deficiency had experienced significant employment difficulties that were resolved once they had access to icatibant at home and at work. The proportion of patients with no HAE-related time off work improved after inclusion in IOS. However, there was no improvement in those with high levels (> 7 days per annum) of HAE-related work absenteeism, suggesting that additional measures are required for this group.

Patients in IOS were managed according to routine clinical practice, providing a comprehensive and an accurate ‘real-world’ description of HAE patients with C1-INH deficiency at three major NHS-funded specialist centres. This prospective registry included only patients who were prescribed icatibant. Factors influencing icatibant prescription have not been formally characterized, but may include previous treatment experience and training, quality of venous access, attack frequency, patient preference, religious beliefs (concerning blood products) and funding decisions (HJ Longhurst, personal communication). These factors may have an impact on the generalizability of the results. In addition, UK data were geographically limited to three large, specialist HAE centres in London, Manchester and Plymouth; therefore, referral bias is inevitable, and data may not be representative of the wider UK experience (UK patients represented approximately 3.5% of the UK HAE population). We believe that continued national commissioning will reduce inequalities of access to treatment and may allow wider access to icatibant in the future.

Other limitations of the analyses, common to most observational studies, include: only patients with available data are analysed; missing data for some endpoints; the variable length of patient follow-up; and potential variability of data collection methodology for attacks occurring prior to IOS entry and during the IOS observational period. In addition, attack severity was described by the patients, therefore, may have been subjective, and a new attack or a relapse of a previous attack were indistinguishable. Furthermore, patients who self-administer icatibant treatment may not follow recommended practice, despite receiving training, and the response to icatibant may vary according to patient practice. The case we described of a patient with very high reinjection and C1-INH rescue use demonstrates that patients’ may develop different treatment practices and highlights that physicians should carefully monitor patients’ use of treatment. However, occasional use of icatibant as a ‘holding’ treatment in patients who routinely use C1-INH for very frequent attacks can, in our experience, be useful in immediately life-threatening situations or where access to C1-INH is likely to be subject to unacceptable delay.

## Conclusions

Data from IOS have helped characterize the experience of HAE patients at three UK centres. Our findings suggest that availability of icatibant treatment may have encouraged patients to become more involved in their care through self-administration, with improvement in some outcomes such as increased rate of treatment for potentially debilitating attacks and HAE-related time off work or study. Compared with their non-UK peers, a higher proportion of UK patients were in work or studying. Those using icatibant reported less time off work than their non-UK counterparts. Ultimately, we believe that HAE management in the UK should focus on the maintenance of wider patient social and physical functioning. Improvements are required to further reduce the unacceptable diagnostic delay and to address the problem of work absenteeism in those requiring more than 7 days off work. The latter may require a ‘whole of life’ approach to disability prevention.

We anticipate publishing a UK IOS update every 3–5 years to help establish benchmarking standards and inform improvements to the health and quality of life in individuals with C1-INH deficiency.

## Abbreviations

AAE: acquired angioedema
BL: baseline
C1-INH: C1 inhibitor
HAE: hereditary angioedema
HCP: healthcare professional
IOS: Icatibant Outcome Survey
IQR: interquartile range
SD: standard deviation
